# Radial Arteriovenous Fistula Formation After Transradial Cardiac Catheterization

**DOI:** 10.7759/cureus.60370

**Published:** 2024-05-15

**Authors:** Barbara Senger, Hassan Eidy, Andrew Gray, Robert Grodman

**Affiliations:** 1 Internal Medicine, Corewell Health Farmington Hills Hospital, Farmington Hills, USA; 2 Cardiovascular Disease, Corewell Health Farmington Hills Hospital, Farmington Hills, USA

**Keywords:** radial arteriovenous fistula, left heart cardiac catheterization, colored flow doppler ultrasound, arteriovenous fistula ligation, cardiac catheterization complications

## Abstract

More than one million cardiac catheterization procedures are performed each year in the United States for both diagnostic and therapeutic purposes. Obtaining access through the radial artery has gained popularity in recent years due to its economic as well as its morbidity and mortality benefits over femoral artery access. However, with any invasive procedure there are associated risks, including arteriovenous fistula formation. This case illustrates the formation of an iatrogenic arteriovenous fistula as a complication of transradial coronary catheterization. Albeit rare, this case will discuss the presentation and clinical course of a patient who was diagnosed with a radial arteriovenous fistula seven weeks post-cardiac catheterization. Ultimately, she underwent direct surgical repair with ligation of the venous branches of the arteriovenous circulation.

## Introduction

In the United States, more than one million cardiac catheterization procedures are performed each year. They are performed with the goal of diagnosing and ultimately treating patients with suspected or confirmed coronary artery disease. Since the first right heart catheterization in 1929, the technique and scope of coronary catheterizations have rapidly evolved [1]. Coronary catheterization is further categorized as either right heart catheterization or left heart catheterization. Right heart catheterizations allow for direct measurement of right-sided cardiac and pulmonary hemodynamics. Left heart catheterizations and coronary angiography provide a diagnostic and therapeutic role in those with coronary artery disease. Left heart catheterization is commonly performed by accessing the radial or femoral artery. Since the first reported transradial approach (TRA) angiogram in 1989, there has been increased popularity due to reduced bleeding risk, increased patient comfort, and lower risk of morbidity and mortality [2]. However, with any invasive procedure, there are associated risks and complications. Vascular complications are not limited to radial artery occlusion or spasm, hematoma, and rare complications including pseudoaneurysm and arteriovenous fistula (AVF) formation [3]. This case reports a rare complication of radial AVF formation and subsequent repair after transradial cardiac catheterization. 

## Case presentation

A 44-year-old African American female with a past medical history of hypertension, hyperlipidemia, alcohol use disorder, and tobacco dependence presented for non-ST-elevation myocardial infarction (NSTEMI) with refractory chest pain. She presented to the emergency department for chest pressure that radiated to her left arm and jaw, which began 24 hours before arrival. Initial electrocardiogram (EKG) showed dynamic ST segment changes with evolving T-wave inversions in the anteroseptal leads. She was taken emergently to the cardiac catheterization lab due to refractory chest pain not resolved with sublingual nitroglycerin or intravenous morphine.

Local anesthetic was administered and access was obtained in the right radial artery using a micropuncture technique and a 6 French Terumo sheath. Therapeutic anticoagulation was achieved with the administration of intravenous heparin. She was found to have a proximal left anterior descending (LAD) artery-filling defect. A drug-eluting stent was successfully placed in the ostial LAD. Immediately after percutaneous intervention (PCI), the arterial sheath was removed. A hemo-band was placed over the arteriotomy site to maintain patent hemostasis. 

**Figure 1:**
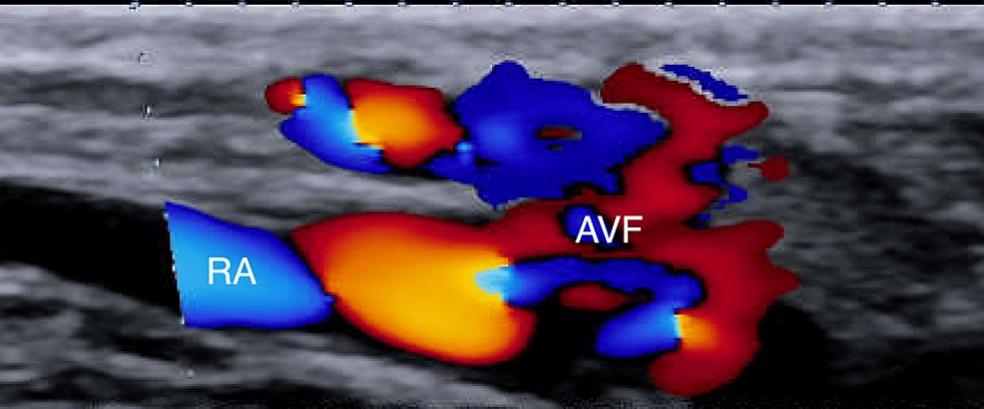
Radial artery with distal bidirectional blood flow indicating arteriovenous fistula formation RA: radial artery; AVF: arteriovenous fistula

**Figure 2:**
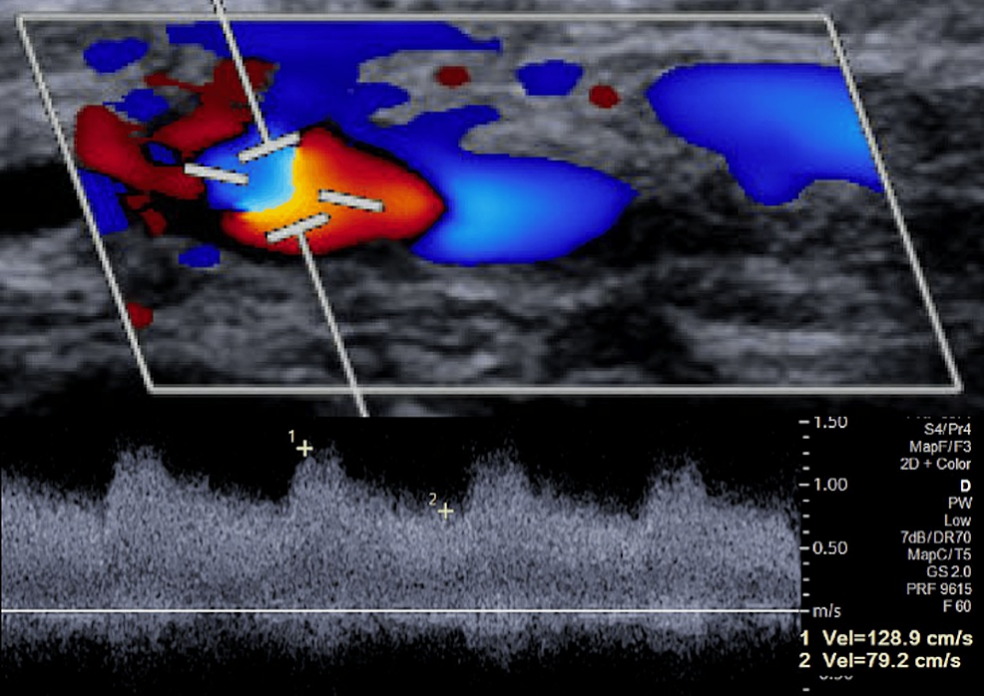
Radial arteriovenous fistula with spectral broadening and elevated systolic and diastolic velocities

The patient was subsequently discharged to cardiac rehabilitation on dual antiplatelet therapy (81 mg aspirin and 10mg prasugrel). Approximately seven weeks post PCI, the patient endorsed mild pain and swelling in her right wrist. She stated, “It feels like there is water running in my wrist.” Upon auscultation, there was a palpable thrill and bruit in the right wrist. An ultrasound with color Doppler of the right upper extremity revealed a right radial artery arteriovenous fistula (Figure 1) with low-resistance blood flow due to the admixed arterial and venous blood flow (Figure 2). 

The patient was referred to a vascular surgeon. Through shared decision-making, the patient opted to pursue surgical intervention. The patient underwent exploration of the right radial artery with ligation of the AVF and repair of the right radial artery. The radial artery was isolated. The venous branches were ligated with 4-0 silk ligature during dissection. After exposing the radial artery, the dissection was carried distally beyond the origin of the deep and superficial palmar arch vessels. The entry site for the PCI access was found to be into the radial artery at the branch of the superficial and deep palmar arch. The repair was performed with primary closure by running a 7-0 polypropylene suture. Closure was hemostatic and sterile dressings were applied.

## Discussion

Left cardiac catheterization procedures require arterial access, with the two most common sites being the common femoral artery and radial artery. Lucein Campeau first introduced the trans-radial approach (TRA) for coronary angiography in 1989, which was later followed by Kiemeneij et al. for percutaneous coronary intervention (PCI) [4]. The TRA has been associated with reduced hospital length of stay, enhanced patient comfort, and overall lower incidence of access site complications when compared to the trans-femoral approach (TFA) [5]. Access site complications are uncommon in the TRA because of the superficial course of the radial artery and the surrounding veins are of smaller caliber in comparison to the femoral artery and surrounding vasculature. In the Radial vs Femoral Access of Coronary Intervention (RIVAL) trial, which included 7,021 patients with acute coronary syndrome undergoing PCI, 0.14% who underwent the TFA developed an AVF compared to none who underwent the TRA [6]. In an additional large case series, by Tatli et al., which included 10,324 patients there were only four cases of AVF formation after undergoing TRA for PCI [7].

Although the TRA has several benefits compared to the TRA, it is not risk-free and both complications may arise. This case demonstrates the importance of recognizing the risk of radial access. Kelm et al. reported several predisposing factors to iatrogenic femoral AVF formation, including age over 60, female gender, arterial hypertension, prolonged coumadin therapy, and high heparin dosage during the procedure [8]. Due to the low incidence rate and limited number of documented cases, including risk factors, it is difficult to speculate if similar predisposing factors can be correlated with iatrogenic radial AVF formation. A literature review found 28 cases of iatrogenic AVF formed after PCI (Table 1) [8-34]. 

**Table 1:** Literature review of iatrogenic radial arteriovenous formation after trans-radial coronary angiography NA: not available; HTN: hypertension; HLD: hyperlipidemia; CAD: coronary artery disease; T2DM: type 2 diabetes mellitus; PVD: peripheral vascular disease; HFpEF: heart failure with preserved ejection fraction; PAD: peripheral artery disease; DVT: deep vein thrombosis; PE: pulmonary embolism

Author	Year	Age	Sex	Symptoms	Cardiac comorbidities	Post PCI AVF diagnosis	Imaging	Intervention	Outcome
Pulikal et al. [9]	2005	64	M	Dilated veins, palpable thrill	NA	5 wks	US	Ligation	NA
Spence et al. [10]	2009	59	M	Mass	NA	1 yr	US	NA	NA
Kwac et al. [11]	2010	67	M	Mass, edema	HTN, Ischemic heart disease	1 yr	US, CTA	Ligation	NA
Yang et al. [12]	2011	54	M	Palpable thrill	Alcohol use disorder, tobacco use	2 mos	US	Ligation	NA
Na et al. [13]	2012	61	F	Palpable thrill, dilated veins	NA	11 mos	US, CTA	(1) Compression ; (2) ligation	(1) Poor; (2) good
Summaria et al. [14]	2012	66	M	Edema, pain	NA	1 yr	US, CTA	(1) Compression; (2) stent	(1) Poor; (2) good
Goldberg et al. [15]	2013	55	F	Palpable thrill, dilated veins	NA	2 mos	US	Ligation	Good
Sugahara et al. [16]	2013	74	F	Mass, pain	HTN, HLD	8 mos	US, CTA	(1) Endovascular balloon-assisted direct percutaneous embolization; (2) conservative; (3) second embolization	(1) Poor; (2) poor; (3) good
Dehghani et al. [17]	2013	62	M	“Swishing” sensation, palpable thrill	No known risk factors for CAD	1 mos	US	Conservative	Good
Dutton et al. [18]	2014	61	F	Mass, pain, palpable thrill, paresthesias along median nerve distribution	NA	2 mos	US	Ligation	Good
Reguerio et al. [19]	2014	56	M	Mass, pain	h/o MI	9 yr	US, CTA	(1) Conservative; (2) Stent	(1) Poor; (2) good
Hashimoto et al. [20]	2015	61	M	Edema, pain	NA	1 wk	US	Compression	Good
Novotony et al. [21]	2016	64	M	Mass, pain	HTN, T2DM	1 yr	US	(1) Embolization x2; (2) Ligation	(1) Poor x 2; (2) good
Nagata et al. [22]	2017	68	M	Mass, pain	NA	7 yrs	CTA	Ligation	Good
Moorthy et al. [23]	2017	62	F	Dilated veins, palpable thrill	NA	3 mos	US, CTA	Conservative	NA
Minhas et al. [24]	2019	58	M	Pain, warmth	HTN, CAD, PVD, HFpEF, T2DM, HLD, h/o tobacco	1. 3 d 2. 3 mos later	US	(1) Conservative; (2) Endovascular embolization	(1) Resolution on Day 4; (4) Resolution
De Oliveira et al. [25]	2019	86	M	Dyspnea, nocturnal hypoxia, palpable thrill	h/o tobacco use, HLD, CAD s/p previous PCI	16 mos	US	Surgery	Good
Shah et al. [26]	2020	71	M	Pain, dilated veins, palpable thrill	CAD, prior PCI, PAD, HTN	1 wk	US	Conservative	Good
Mehta et al. [27]	2020	74	M	Paresthesia	HTN, HLD, T2DM	1 d	US	Conservative	Good
Herzallah et al. [28]	2021	85	M	Dyspnea, pain	HTN, HLD, recurrent DVT/PE	2 mos	US	Surgical ablation	Good
Kelm et al. [8]	2021	71	M	Dilated veins, palpable thrill	CAD, PAD, HTN	1 wk	US	Conservative	Good
Dakik and Haddad [29]	2022	56	M	Mass	Bicuspid aortic valve	6 wks	US	Surgical intervention	NA
Gu et al. [30]	2022	73	F	Paresthesia in the median nerve distribution, edema, palpable thrill	NA	1.2 wks 2.6 mos later	US, CTA	(1) Compression; (2) ligation	(1) Poor; (2) good
Maeba et al. [31]	2022	71	M	Pain, cold sensation	HTN	3 mos	US, CTA	(1) Compression; (2) ligation	(1) Poor; (2) good
Okam et al. [32]	2023	51	F	Intermittent paresthesia	Prior MI s/p PCI	Preceding mos	US	Conservative	NA
Okam et al. [32]	2023	72	F	Palpable thrill	NA	2 yrs	US	Ligation	NA
Petrochko et al. [33]	2023	42	M	Mass, pain	Tobacco use	22 mos	US, CTA	Ligation	Good
Mahanta et al. [34]	2024	45	M	Warm sensation, pain, edema	Tobacco use, alcohol use disorder	6 mos	US	Ligation	Good

There is a 2.5:1 male-to-female predominance with an average age of 63.9 years [8-34]. A majority of those who form an iatrogenic radial AVF do not develop significant symptoms that cause hemodynamic compromise. The clinical manifestations are usually related to venous dilation as the arterial blood bypasses the capillaries. The most common presenting symptoms were 46% presenting with pain (n=13), 42% with a palpable thrill (n=12), and 39% with edema (n=11). Comorbidities and cardiac risk factors are only specified in 17 of the 28 case reports with 47% having underlying arterial hypertension (n=8), 29% who either currently or have a history of tobacco use (n=5), 29% with hyperlipidemia (n=5), and 18% who have undergone previous PCI (n=3) [8-34]. In this case, the patient was a 44-year-old female with several comorbidities that may contribute to her risk of developing the fistula. Her presenting symptom was an abnormal sensation in her right wrist seven weeks post-cardiac catheterization. 

Duplex ultrasound imaging is usually the initial diagnostic tool of choice to evaluate the site and grade of the fistula due to it being readily available, low cost, and non-invasive. Characteristic ultrasound findings will demonstrate low resistance blood flow in the anastomosed artery. The anastomosis site will also show turbulence and high-velocity blood flow [35]. If ultrasound is inconclusive, a computerized tomography angiogram (CTA) may also be performed to further evaluate the blood flow through the fistula, as well as to further delineate the exact location of the fistula. Most studies opted solely for utilizing ultrasonography to diagnose the AVF 64% (n=18), 32% utilized both US and CTA (n=8) to evaluate the fistula, while only one case (n=1) underwent CTA [8-34]. In this case, only US was used for the diagnosis of the fistula, demonstrating a broadened, low-resistance waveform seen in the brachial and radial arteries, as well as at the arterial anastomosis (Figure 1). 

Management of the fistula depends on several factors, including patient symptoms and presence of hemodynamic compromise. The goal of treatment is to isolate the anastomosis and close the fistula while maintaining blood flow. Fistula repair can be performed using several methods including conservative therapy, primary repair via ligation or with a graft/bypass, or through endovascular repair. Surgical ligation had the highest success rate, including the patient in this case, with complete resolution of the fistula. However, not all patients who develop an iatrogenic fistula need to undergo surgical intervention, especially since this patient population likely has several comorbidities which may make them poor surgical candidates. Several patients also had good outcomes with conservative therapy alone [8,17,20,26,27]. 

Although the development of an AVF is a rare adverse event of the TRA of PCI, it is important to remain cognizant. The incidence can be further minimized with operator experience, limiting the number of times the artery is accessed, smaller sheath size, and using ultrasound-guided needle placement. 

While there have been studies evaluating the risk factors associated with iatrogenic femoral AVF after PCI, there have not been enough cases of iatrogenic radial AVF to assess if similar risk factors apply in those undergoing TRA for PCI. In this case, the patient shares two risk factors of female sex and arterial hypertension. It is speculated that women have a higher risk of AVF due to smaller vascular diameter which could make it more difficult to achieve access which in addition to having an increase in vascular stiffness from underlying hypertension may have contributed to this patient’s radial AVF formation. Further contributing factors that may have led to this patient developing an AVF include the lack of ultrasound-guided arterial access and the several other cardiac risk factors the patient had, including hyperlipidemia, alcohol use disorder, and tobacco dependence. Being aware of and mitigating these potential risk factors can only further minimize fistula formation and a successful procedural outcome. 

## Conclusions

Iatrogenic arteriovenous fistula formation is a rare complication of TRA of PCI. While often benign, the fistula may provide significant discomfort to patients. Patients thus often choose to undergo surgical intervention to fix the vasculature abnormality. Since there are relatively few published cases of iatrogenic radial AVF from PCI it is unclear whether similar risk factors for femoral fistula formation apply. Nonetheless, duplex ultrasound is a quick, non-invasive tool which can be utilized to allow for direct visualization of the anatomy during catheter placement to further avoid AVF formation.
